# Supplementary material for: Defects in G-Actin Incorporation into Filaments in Myoblasts Derived from Dysferlinopathy Patients Are Restored by Dysferlin C2 Domains
Source: Int J Mol Sci. 2019 Dec 19;21(1):37. doi: 10.3390/ijms21010037 (PMC6981584; doi:10.3390/ijms21010037)
Supplement: Supplementary file 1 [file ijms-21-00037-s001.pdf]

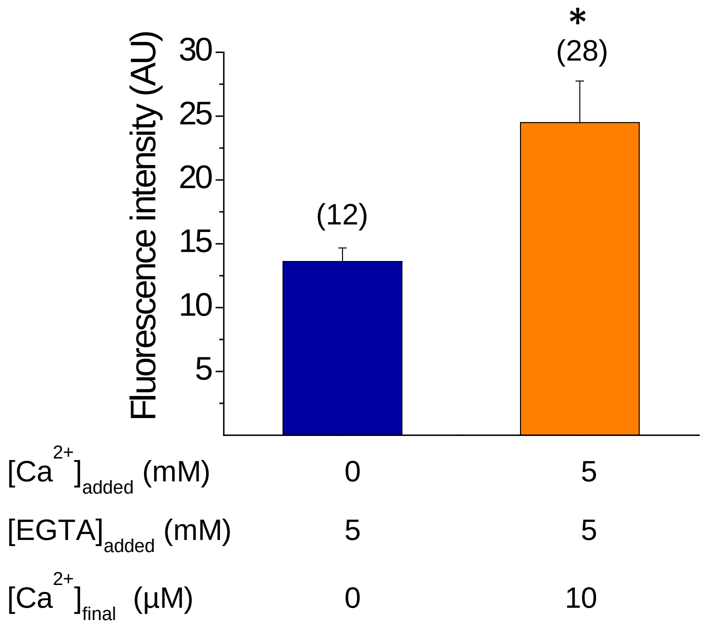

Figure S1. *High Ca^{2+} concentrations increase G actin incorporation into filaments*. Fluorescently-tagged G-actin incorporation into filaments was assayed in myoblasts permeabilized with 20 µM digitonin during 6 min at 37°C, in the presence of 300 nM Alexa-Fluor-488 actin and 2 mM ATP-Mg^{2+}, in the absence or presence of free Ca^{2+} (10 µM). Concentrations of Ca^{2+} and EGTA were estimated using the Ca-EGTA Calculator v1.2 program (University of California, Davis, USA). Actin fluorescence intensity was measured in a single focal plane, and normalized by the cell area. Images were analyzed using the ImageJ software (NIH, USA). Data represent means ± SEM. The number of analyzed cells from five different cultures is indicated in parentheses. *$p < 0.05$ (t-test).

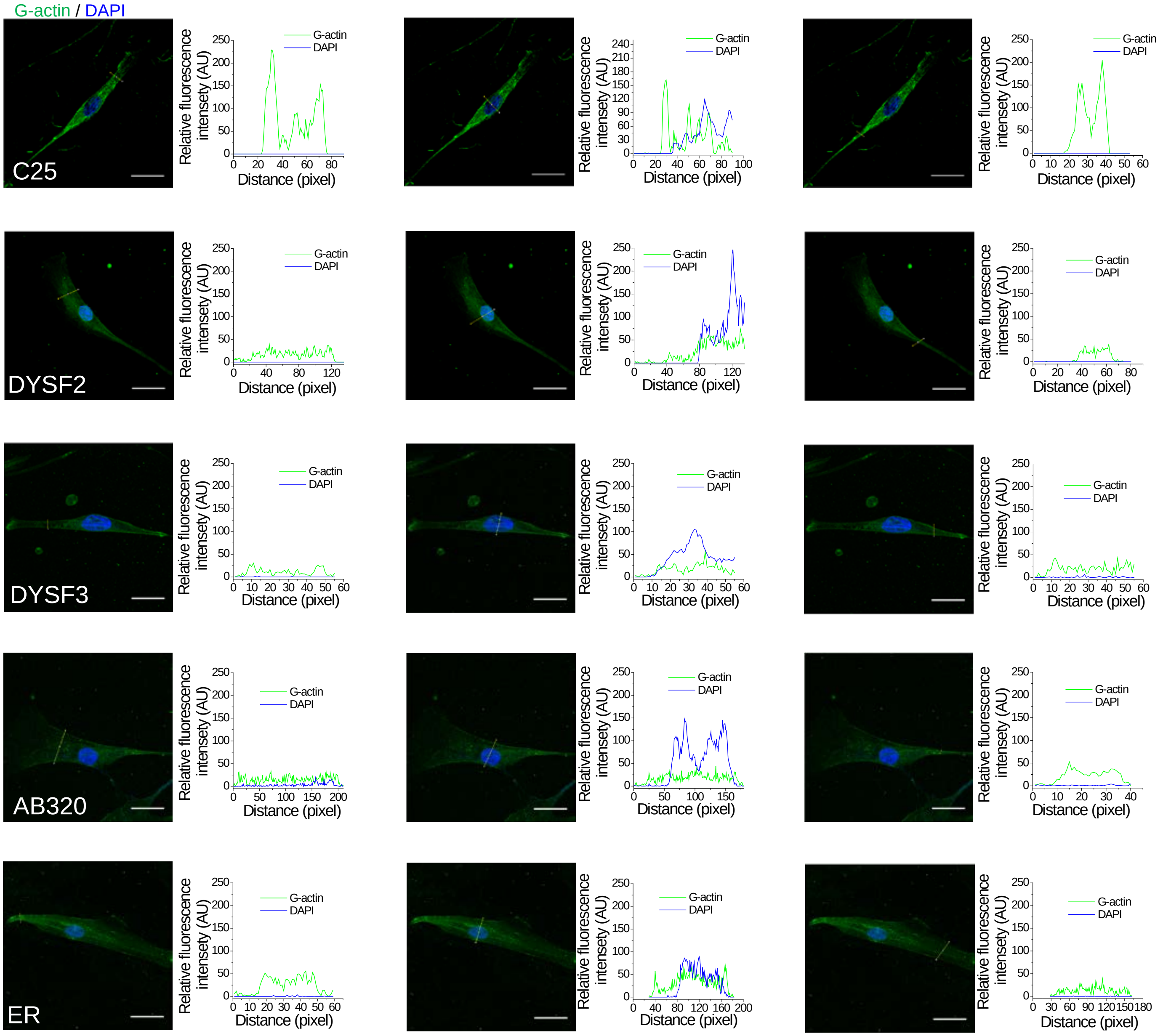

Figure S2. *Intensity profiles of G-actin in C25 and dysferlin-deficient myoblasts*. The images show merges of Alexa-Fluor-488 actin (green) and DAPI (blue) from control C25 and dysferlinopathy DYSF2, DYSF3, AB320 and ER myoblasts shown in Figure 3A. The graphs show 1D intensity plots obtained using the ImageJ software (NIH, USA). Three regions per cell were analyzed that are shown with dotted white lines in the images.

Table S1. *Incorporation GST- amphiphysin-1 SH3 domain fusion protein in permeabilized C25 and DYSF3 myoblasts*. C25 and DYSF3 myoblasts were permeabilized with 20 µM digitonin in KGEP buffer containing of GST- amphiphysin-1 SH3 during 6 min at 37°C. Afterwards, cells were fixed with 4% p-formaldehyde and incubated with a monoclonal GST antibody (1:50; Invitrogen, Carlsbad, CA, USA) and a secondary CY2-conjugated goat anti-rabbit IgG antibody (1:250; Jackson Immunoresearch, West Grove, PA, USA). Images were captured using a 40X objective in an inverted microscope (Eclipse Ti-E, Nikon, Tokyo, Japan). Images were analyzed using the ImageJ software (NIH, USA). Non-significant differences were found ($p>0.05$; t-test).

Cell line	Relative fluorescence (A.U.)	Number of analyzed cells
C25	841.9 ± 47	40
DYSF3	757.5 ± 18	44

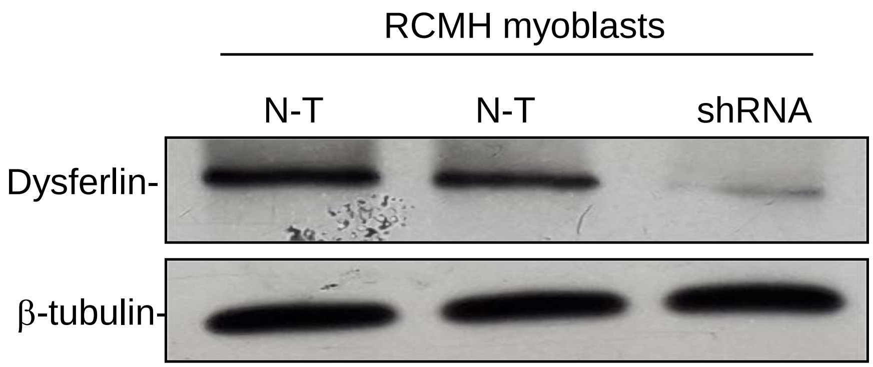

Figure S3: *Dysferlin expression in RCMH cells with stable dysferlin knockdown*. Dysferlin expression was determined by immunoblotting of total protein extracts from RCMH myoblasts non-transfected (N-T) or stable transfected with shRNA against dysferlin (Santa Cruz Biotech, Dallas, Tx) using an anti-dysferlin-HAMLET antibody (Novocastra TM Lyophilized Leica; Newcastle, United Kingdom). An anti-β-tubulin antibody (Cytoskeleton, St. Denver, CO, USA) was used as a loading control (bottom bands).

Table S2: *mCherry fluorescence intensity in C25 and DYSF3 myoblasts*. C25 and DYSF3 myoblasts transfected with mCherry, or dysferlin N-terminal (N-term) or C-terminal (C-term) fused to mCherry were visualized by confocal microscopy, and images were captured at the equatorial plane of the cells using identical exposure settings between compared samples. Images were analyzed using the ImageJ software (NIH, USA). Data correspond to mCherry fluorescence intensity corresponding to data from the indicated figures.

Figure	Construct	Cell	Fluorescence intensity (AU) Mean ± SEM	Number of cell
Figure 3e	mCherrry	C25	41.5 ± 5.1	19
Figure 3e	N-term	C25	18.8 ± 6.1	20
Figure 3e	C-term	C25	18.7 ± 2.3	22
Figure 3e	mCherry	DYSF3	52.7 ± 4.5	22
Figure 3e	N-term	DYSF3	13.0 ± 2.1	15
Figure 3e	C-term	DYSF3	20.2 ± 2.2	21
Figure 5d	mCherrry	DYSF3	11.8 ± 2.7	45
Figure 5d	N-term	DYSF3	13.7 ± 3.6	22
Figure 5d	C-term	DYSF3	11.9 ± 9.7	45

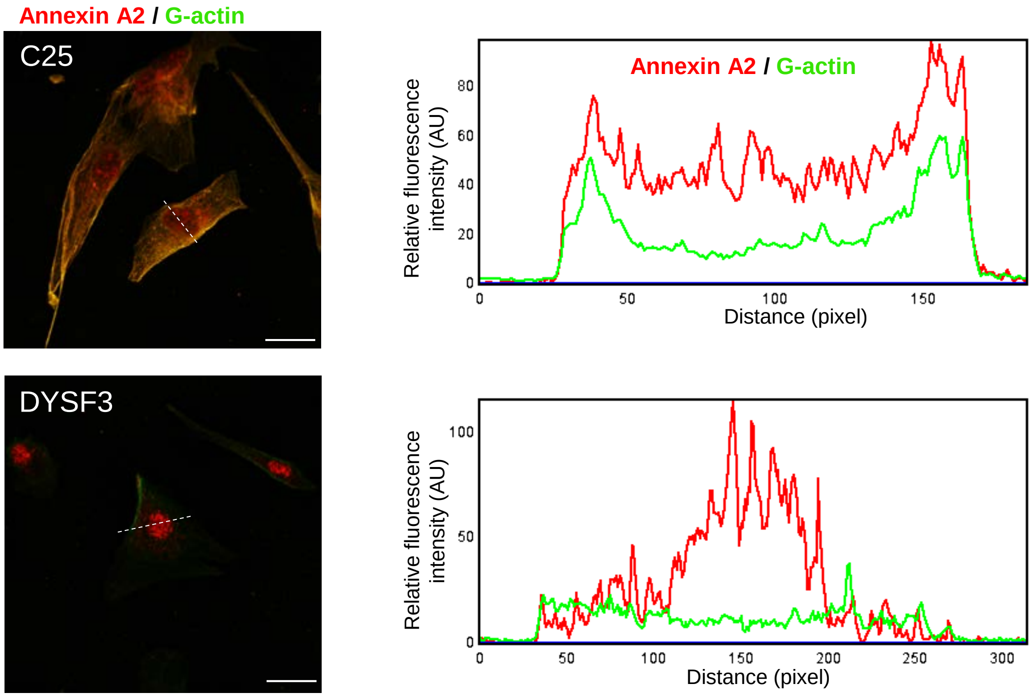

Figure S4: *Intensity profiles of annexin A2 (red) and G-actin (green) in C25 and DYSF3 myoblasts*. The images show merge of annexin A2 and Alexa-Fluor-488 actin from control C25 and dysferlinopathy DYSF3 myoblasts shown in Figure 5A. The graphs show 1D intensity plots obtained using the ImageJ software (NIH, USA). The dashed white lines in the images show the analyzed regions.
